# Editorial: Global Health and Pharmacology

**DOI:** 10.3389/fsoc.2021.765172

**Published:** 2021-09-28

**Authors:** Susan Leigh Craddock, Dominique Tobbell

**Affiliations:** ^1^ University of Minnesota Twin Cities, St. Paul, VA, United States; ^2^ University of Virginia, Charlottesville, VA, United States

**Keywords:** pharmacology, global health, access, inequity, drug pricing, health insurance

## Troubling Access

Across the many issues pertaining to pharmaceuticals worldwide, ‘access’ is a common denominator and a critical facet of what makes pharmaceuticals life changing, heartbreaking, deeply problematic, dangerous, complex, and successful. As such, in this issue, we seek to critically appraise what constitutes access in the arena of global pharmaceuticals. Over the last few decades, for example, human rights campaigns have pushed for access globally to affordable antiretrovirals and for the development of drugs for neglected diseases. Both of these in different ways made visible the increasingly key role of pharmaceuticals in defining our lives and what constitutes health; the difficulties of achieving equitable geopolitical governance of pharmaceutical development and distribution; and the differential valuation of human lives made evident by the presence or absence of essential medicines and vaccines. While access usually is invoked as a positive, however, it also carries its own risks. Access to too many pharmaceuticals because of pharmaceutical industry efforts to significantly expand markets for old and new pharmaceuticals is the example most often noted in the media and scholarship. But other kinds of problematic access also need further interrogation, such as access to the wrong kinds of pharmaceuticals including counterfeit, contaminated, or substandard; the predominance of biomedical over so-called alternative medicines; and the highly controversial means, such as bioprospecting, that sometimes characterize the discovery, development, and control of new drugs. All of these and other facets of access can wreak serious havoc with public and personal health, economies and ecologies, global economic and political relations, productions of knowledge, and regulatory systems.

The COVID-19 pandemic brought with it its own set of access issues, some a consequence of the urgency that pandemics mobilize, some unique to the disease, and others, accentuations of longer standing issues. In the early days of covid for example there were highly politicized discussions over the efficacy, or not, of potential treatments for covid such as the malaria medication hydroxychloroquine. On the one hand the then-US President Trump touted the role of hydroxychloroquine in curing his own case of covid, while scientists and public health officials decried Trump’s advocacy for a drug that did not have rigorous evidence from clinical trials affirming its success in treating SARS-CoV-2. As the Brookings Institute aptly pointed out in a subsequent report, the collective responses to hydroxychloroquine not only exemplified the challenges of how various publics weigh health risks versus action in times of fear and uncertainty; it also made clear that under situations of crisis the very definitions of ‘adequate evidence’ and ‘science’ become unsettled. As the authors summarize, “While the hydroxychloroquine story is sometimes viewed as a battle between legitimate scientific information vs. dangerous misinformation, this fails to consider debates within medicine about when new evidence reaches the level of ‘actionability’” (Khorana and Owens 2020). Such decisions over safety and efficacy of vaccines or drugs versus urgency in the face of high morbidity and mortality have been seen with other epidemics as well, including HIV/AIDS treatment activist’s calls in the early 1990s for using “surrogate markers” of treatment efficacy rather than mortality data to get drugs developed and approved more quickly (Epstein, 1997), or more recently, a rapidly developed vaccine for Ebola.

As the world watched covid spread from China to the EU to the US, the inextricable interrelations of medical treatments, global supply chains, and the strength of health care and public health systems became more glaringly obvious. As skyrocketing cases of covid overwhelmed one health system after another in high-income countries proud of their state-of-the-art facilities, the underlying fragility of such systems in the face of machine, technology, and ingredient shortages became all too apparent. Though ventilators, PPE, and oxygen are not technically pharmaceuticals, they play parallel roles in being ensconced within health care systems, keeping people alive, and highlighting the broader array of facets beyond patents and prices involved in making available critically important tools. In the case of ventilators, it laid bare the inequities across hospital facilities in affording expensive equipment for their patients, and the too-frequent reality that where you live determines whether you get to live. In the case of both ventilators and PPE, it evidenced the role of governments in incentivizing manufacture of needed equipment, such as the US’s Defense Production Act signed by Trump to galvanize production of PPE by the military (Soucheray, 2020) and by companies like General Motors. It attested as well to the flexibility of global companies in adapting their commodities to pandemic need. Yet, the ugly underbelly of global capitalism also came to the forefront as some of these same companies profited handsomely from emergency sale of medical equipment while paying their workers suboptimal wages and failing to provide them with any of the PPE they were manufacturing and exporting. As they helped save lives in other parts of the world with their protective equipment, they put the lives of thousands of their own workers at higher risk (Beech, 2020).

The role of intellectual property regimes during Covid also, not surprisingly, came to the forefront as attention turned toward pharmaceutical technologies with the potential to staunch exploding caseloads across wide swaths of the globe. On the one hand there were many positive examples of activism towards greater biomedical collaboration in the face of pandemic. Scientists and scientific journals started keeping channels of knowledge sharing open; a longstanding student organization, Universities Allied for Essential Medicines (UAEM), created an online dashboard tracking funding levels of institutions and countries for pharmaceutical R&D (Myhr, 2020); and calls were issued from multiple agencies including the Medicines Patent Pool and Unitaid for creating or expanding patent pools to expedite research and development of potential treatments, medical equipment, and diagnostics (Thievenaz, 2020; ‘t Hoen, 2020). Yet calls for changes in the way pharmaceutical development and distribution happen were met with pushback at the corporate, national, and transnational levels. South Africa’s call for a waiver for some provisions of the TRIPS Agreement in the midst of Covid, for example, was opposed by most higher income countries including those with robust pharmaceutical industries. Those joining South Africa were almost entirely low-income countries, signaling a geopolitical division all too often seen in questions of pharmaceutical access, and a division that remains curiously under-scrutinized (Balasubramaniam, 2020). Pharmaceutical and biotech companies also reacted variably to the calls for knowledge sharing. As Ellen ‘t Hoen of the Medicines, Law, and Policy organization notes, Abbvie waived patent rights to one of their candidate drugs for treating Covid (2020) while also joining public-private partnerships like ACTIV - a collaboration of the NIH, regulatory agencies, the CDC, academics, philanthropies, and biopharmaceutical companies—in order to expedite development of Covid treatments and vaccines (nih.gov). Gilead, on the other hand, convinced the FDA to ascribe Orphan Drug status for remdesivir in a move to strengthen their proprietary rights over the only antiviral medication proven to shorten recovery times for those hospitalized with Covid and approved by the FDA (‘t Hoen 2020; NIH 2020). Only following a huge outcry did Gilead remove remdesivir from orphan drug status (‘t Hoen 2020).

Vaccines have been the latest battle ground for issues of equity in pharmaceutical access under Covid, the pandemic bringing renewed urgency to an old and persistent issue. Despite attempts early on to create tools for addressing the highly unequal distribution of vaccines, low-income countries have once again been relegated to the back of the line. In the absence of progress in TRIPS waivers or new policies, the Global Vaccine Alliance (GAVI), the Coalition for Epidemic Preparedness Innovations (CEPI) and the WHO came up with COVAX, a voluntary mechanism for pooling available vaccine doses and subsequently serving as the common market through which countries could purchase doses for their populations. Though many countries bought in to COVAX, those that could (i.e., the US, EU, and United Kingdom) also prepurchased the vast majority of Covid vaccines while they were still in the pipeline. As has happened before, national responsibilities quickly eviscerated any moral obligation towards equitable global rollout, despite the logic that geographic hoarding will only delay the end of the pandemic. In other words, whether in 2009 H1N1 flu or COVID, the US and other high income countries first took care of their own populations before donating leftover doses of vaccine to lower income countries (Enserink 2009; Craddock and Giles-Vernick 2010). Renewed calls for requiring patent waivers have gained some traction, even seeing President Biden get on board with the idea; yet as others have noted (Schellekens 2021), removing patents will not fix the problem when chronically underfunded public health infrastructures, inadequate health personnel, inability to sustain cold chain storage in the face of frequent electricity failures, and other issues translate into huge challenges in getting vaccines into bodies even if they got into more countries.

The call we put out for this Research Topic predated the pandemic, and therefore our contributors do not specifically address the many facets of global pharmacology galvanized by SARS-CoV-2. Yet as is evidenced by the papers we received, Covid-19 has highlighted many issues in pharmaceutical research, development, marketing, and distribution that are not new, but whose possibilities, deficits, and constraints are newly spotlighted in the midst of urgency. Our collection reminds us that we need to valorize those aspects that are working in our public health and health systems, while heeding those facets that remain problematic even when our heightened awareness fades post-pandemic.

In our call for papers, we solicited scholarship on facets of access that are new and underexplored, as well as scholarship that revisits issues, such as access to ARVs, that have largely faded from our collective attention even while still all too trenchant in many parts of the world. We also encouraged empirical studies focused on particular regions, as well as more theoretical and conceptual treatments of past, present, or future interventions in the relationship between pharmaceuticals and global health. We encouraged an array of disciplinary perspectives across the sciences, social sciences, and humanities, as well as multi- or transdisciplinary scholarship. Our goal with this volume was to trouble the very concept of access, raising critical questions about the processes through which access assumes meaning, is circumscribed, contested, and politicized; for whom, under what conditions, and for what purpose. Our goal was to offer insights into past and current global health interventions while also raising possibilities for future policy changes in the ongoing crises of pharmaceutical access. What we got was a rich array of articles examining pharmaceutical access along three broad thematics: multidimensional facets of consumption; the relations between policies and access; and relations between health care systems and access.

## Facets of Consumption and ‘Drugs Into Bodies’

The first category recognizes that access extends beyond the existence or availability of drugs and into areas of how and whether drugs get into relevant bodies. Consumption politics and practices matter to policy analysts and government health officials tasked not just with whether particular pharmaceuticals are available in their respective countries, but what factors determine how and whether individuals are able or willing to consume them. As the articles in this section attest, these factors have a wide range encompassing the media, practitioner expertise, increasing contentions over the pharmaceuticalization of health (cf Biehl 2007), drug use guidelines and oversight, and disease awareness and understanding.

Mekuria and othercolleagues look at self-medicating practices among college students in Amhara, Ethiopia. What they find is that 68% of the students they surveyed practice self-medication, and that pharmacies were the most common places where they obtained medication. Though the pharmaceuticals used without expert guidance were for minor ailments such as headaches, the authors point out the dangers involved in the practice of self-medicating overall—the potential for adverse reactions, drug resistance, treatment failure, drug dependence, and waste of resources prime among them. Though the article did not go into policy recommendations, it seems clear that pharmacists might be targeted for training individuals in how and when to use pharmaceuticals obtained from their pharmacies.

Tousignant’s ethnography of hepatitis B virus (HBV) infection and care in Senegal reveals a reversal of the typical lack of drugs in a low-resource setting to treat high burdens of disease. Instead, Tousignant uncovers complex interplays between low levels of knowledge of HBV even among many practitioners, inadequate screening of the virus, and high cost and urban concentration of diagnostic technologies - all in the midst of general availability of tenofovir to treat against potential development of liver cancer. Tousignant’s fascinating analysis troubles low consumption of an effective and accessible drug by questioning, *inter alia*, the many reasons behind HBV’s relative invisibility even when most public health officials estimate high burdens of infection. The politics of international funding that not only target specific diseases rather than broader facets of public health, and that have for some time now privileged HIV/AIDS, is part of her answer.

Ortega and Müller article on ADHD in Brazil remind us that pharmaceuticals play a differently controversial role in many mental health issues including ADHD—namely, that ADHD is debated at both a global and national level as to whether it is a diagnosable illness best managed through pharmaceutical treatment, or a social-cultural construction. The former contingency, including the Global Mental Health movement, claims that a treatment gap exists in low- and middle-income countries (LMICs) which needs addressing, while the latter contingency claims that this position aids the expansion of the pharmaceutical industry unnecessarily while ignoring differences in interpretation of what constitutes acceptable ranges of behavior. They argue, too, that the literature on this topic has not taken into account the many variations across national settings that themselves encompass both camps and the tensions thereof. Brazil is no exception, and Ortega and Müller provide a meticulous examination of the powerful sway of psychiatry and the argument for treating ADHD on the one hand, and the activism of many who stridently oppose the medicalization of childhood and the imposition of western-centric biomedical models of behavior. Consumption of pharmaceuticals prescribed for ADHD, then, becomes a political stance as well as a contested method of behavioral engineering.

## Relations Between Policy and Access

Exorbitant prices for drugs in the US have become increasingly visible and controversial over the last 2 decades or more. Patents, too, came into the spotlight more than twenty years ago for their critical role in keeping prices high and consequently denying essential medicines to those without the means, government subsidies, or insurance to afford them. Antiretrovirals entering the market, but out of reach for the vast majority of those living with AIDS, catapulted access to medicines into a global movement and a human rights issue. But high prices and patents are not the only facets shaping access to medicines, diagnostics, and vaccines around the world. Various policies from a multitude of sources can also play vital roles in shaping who is able to obtain life-saving or life-improving pharmaceuticals, and who is not; and whether the access constraints are from high prices, or from other deterrents. As the articles in this section demonstrate, these policies are emanating from governments, global organizations, and pharmaceutical companies themselves, and each contribution attests to the importance of discerning the mechanisms through which parameters of access are determined, what the supporting architectures of those mechanisms are, and who is included vs. excluded.

With his case study of Novartis’s Zolgensma, Sergio Sismondo extends important scholarship on the ways in which pharmaceutical companies navigate a balancing act between sky high new drug prices, public condemnation, and accessibility around the globe. At $2 million for a one-time gene therapy treatment for spinal muscular atrophy, it would seem that Novartis arrived at a price no one could afford. Sismondo walks readers through the entangled landscape of insurance structures, the global health access to medicines movement, corporate social responsibility schemes, and the WHO’s statement on the rights of citizens everywhere to essential medicines and the ethical impetus on pharmaceutical companies to ensure that. One of the critical moves Sismondo makes in this piece is to not take at face value the existence of global movements or WHO essential medicines lists, but rather to uncover how and why these entities come into being and gain traction if not institutional normalization. And as they do gain traction, pharmaceutical companies like Novartis become adept at outmaneuvering their reach through a combination of language appropriation and public relations policies. In Sismondo’s hands, policies, guidelines, and movements form a highly dynamic and constantly shifting terrain where pharmaceutical companies, not publics, are the winner.

In her article on changing pharmaceutical governance in China, Li elucidates the multiple facets constituting access to medicines, from availability, accessibility, appropriateness, and affordability, while also questioning the contradiction and relations of these facets as they reside uneasily within national health policies. As she suggests, few scholars have historicized and deconstructed how and why particular countries shift their policies over time to focus on one facet or another within the access to medicines arena. More specifically, Li argues that not only are these various dimensions often in tension with each other, but the reasons for a country to shift prioritization of one over another facet can involve social, economic, political, or other contexts. Taking China as her case example, Li proceeds to incorporate macro-level (political economies of pharmaceutical and health sectors) and micro-level (organizational, cultural, and community) analyses of why the Chinese government shifted focus from availability, to affordability, or both, across multiple decades beginning in 1949 with Mao’s ascension to power. In those earlier days drugs were affordable, yet they were scarce given levels of economic disruption from war and poverty. Availability of drugs was thus the focus through the 1970s, with various government initiatives geared toward expanding pharmaceutical industry capacity while simultaneously strengthening health sector efficiency. With economic liberalization and the collapse of centralized payment schemes in the 1980s came greater availability yet lower affordability for drugs, mobilizing new efforts to drive down prices through various policies from establishing an essential medicines list to creating procurement schemes. In the last 6 years, China has struggled towards a balance between both affordability and availability as it faces continued high prices, shifting demands for greater coverage of cancer and chronic diseases, and a need for greater innovation within the pharmaceutical industry.

Atuk turns toward a specific pharmaceutical company, Gilead, and one of their particular drugs, Truvada (known as PrEP), to provide an exploration of pathopolitics as it is playing out with gay populations in the US in the age of HIV/AIDS. As Atuk explains it, pathopolitics is biopolitics as practiced by pharmaceutical companies that address certain pathologies while simultaneously producing new ones. More specifically, pharma’s relentless quest to find new markets for drugs means creating new categories of risk, which in turn demand pharmaceutical investment. This capitalization of bodies, as Atuk argues, keeps pharmaceutical companies healthy while pathologizing those bodies and populations as perpetually at risk—in Atuk’s case, of HIV/AIDS. So, antiretrovirals for treating AIDS are no longer sufficient; Truvada has come to stand as a requisite supplement for gay men constantly at risk of, but wanting to avoid, HIV. And yet, significant populations of gay men are left out of this chance at warding off HIV because of Truvada’s price tag. That results in more literal pathologies as those hundreds of thousands most at risk of HIV in the US, namely gay men of color and transgender women, are the least able to afford Truvada and thus acquire HIV through lack of ‘PrEParation.’ Atuk subsequently traces the paths of pharmaceutical violence against these marginalized populations, invoking Rob Nixon’s concept of slow violence (Nixon, 2011) in elucidating those declines and death that are neither spectacular nor particularly visible, but that are nonetheless violent because they are preventable, and occur through the exacting calculations of profitability. The bottom line, as Atuk argues, is that “human life is protected only insomuch as it promises financial returns.”

Cappello et al. examine the history and contemporary implications of the ‘square box’ concept used in the World Health Organization’s Model List of Essential Medicines. The square box, which was first introduced in 1983 (5 years after the first Model List was introduced) designates a representative medicine in a group of equivalent and interchangeable medicines. It is intended “to highlight pharmacological classes or groups of medicines for which countries, institutions, and health professionals can assume homogenous therapeutic efficacy and safety and select the most appropriate single medicine based on price, local availability, and acceptability.” (p. 1). Collectively, the Model List and square box concept are intended as a mechanism for increasing access to essential medicines. However, as Cappello et al. note, many countries find it difficult to navigate the utility of the Model List including how to make best decisions concerning their national pharmaceutical needs and budget, versus what is listed within the Model List. These authors then don’t just critique the square box concept but seek to clarify both the interpretations and the practical applications of the Model List so that countries may better arrive at appropriate pharmaceuticals for their national needs. They pay particular attention to the distinctive problems presented by biologic medicines, which tend to come with very high price tags and for which generic equivalents do not exist. Instead, there are biosimilar medicines, which as their name implies are highly similar to the original biologic medicines and which are approved by regulatory agencies to be manufactured when the original patent product expires. Biosimilars are neither identical nor bioequivalent to the original innovator product, which in turn raises another set of questions about whether and how to apply the square box concept to biologic essential medicines. In addressing these questions, Capello and others make clear the promise but also the limitations of any one-fits-all tool for navigating such a complicated arena.

Shahriar and Alpern problematize the effects—intended and otherwise—that the Food and Drug Administration’s policies have had on prescription drug prices in the U.S. by analyzing the pricing story of two antiparasitic essential drugs. The antiparastic drug market is an example of a relatively low-volume drug market which, the authors argue, can “become incubators for opportunistic manufacturer behavior.” In their first example, albendazole, which has been marketed outside the US since 1982 and was FDA approved in 1996, Shahriar and Alpern document the pharmaceutical industry practice of instituting price-hikes on old, off patent essential drugs. In this case, a series of corporate acquisitions of albendazole’s manufacturers led to delayed entry of generic albendazole and enabled monopoly-like conditions on the drug which allowed sequential and significant price-hikes. Shahriar and Alpern suggest, however, that the FDA’s policy, since 2017, to incentivize generic entry in non-competitive markets finally led generic manufacturers to enter the market in September 2018. However, a significant reduction in average wholesale price for albendazole—anticipated when generic competitors enter the market - is yet to materialize. The author’s second example, in contrast, problematizes a different FDA policy. The tropical disease priority review voucher program, introduced by the FDA in 2007, was intended to incentivize research and development for neglected tropical disease drugs. But as Shahriar and Alpern show using the example of miltefosine, rather than lead to the development of new and innovative drugs, the voucher program instead helped drug firms bring already-existent drugs to the U.S. market and to make profits from them, rather than using the voucher to prioritize new, and very much needed, drugs. In addition to raising the alarm about the unintended pricing effects of FDA policies, Shahriar and Alpern also offer recommendations for closing the loopholes and limitations of those policies.

Suh examines the contested role of misoprostol as an essential reproductive health medicine in sub-Saharan Africa, a region with some of the highest rates of maternal mortality and fertility in the world. Misoprostol is safe and effective as an off-label treatment for post-partum hemorrhage, post-abortion care, and the provision of first trimester pregnancy termination. However, its potential as an abortifacient complicates misoprostol’s status as an essential obstetric medicine. In her essay, Suh analyzes the ways in which the WHO, national and international NGOs, funding organizations like the USAID, and philanthropic agencies have negotiated misoprostol’s abortifacient qualities and integrated misoprostol into reproductive health policy and practice. She does so in the context of the region’s restrictive abortion laws, institutionalized abortion stigma, the anti-abortion policies that govern USAID’s work, the imperatives to pharmaceuticalize global health, and the long history of (neo)colonial population governance in sub-Saharan Africa, making clear that having a drug listed as an essential medicine is not sufficient to guarantee access. Although community-based health workers, including traditional birth attendants, can safely administer misoprostol, the continued privileging of neoliberal health reform, Suh argues, significantly undermines access to misoprostol. Indeed, the privatized distribution of misoprostol in health facilities and pharmacies reinforces the marginalization of community-based health workers upon whom low-income and rural women often depend for maternal and reproductive health care. Suh’s analysis thus highlights the influence of the region’s complex transnational politics on shaping how and where misoprostol is available and used (or not), and by whom. In doing so, Suh makes clear both the benefits and limits of pharmaceuticalizing reproductive health, not least the inability to secure reproductive justice for low-income and rural women seeking access to safe and effective reproductive care in SSA.

## Relations Between Health Care Systems and Access

The third category recognizes that the ways in which governments organize, fund, manage, and deliver care within a health system has a determinative impact on individual and community access to pharmaceuticals. Whether governments finance, manage, and deliver care through centralized national health systems or utilize a mix of public and private sector financing and provision, different types of health systems and the political economies that underpin them lead to different opportunities for, and barriers to, access. Publicly financed provision of pharmaceuticals, for example, is rarely sufficient to guarantee access to pharmaceuticals; there also need to be enough health care facilities and workers with prescriptive authority to provide and ensure proper consumption of pharmaceuticals in the communities that need them. In health systems that rely on private health provision, individuals may face prohibitively high out of pocket expenditures for prescription drugs because they are either uninsured or their insurance does not provide adequate coverage for the drugs they need. In such cases, individuals may either forego filling their prescriptions or ration their use of life-saving medicines, which in turn can result in preventable deaths and unnecessary morbidity. In 2015 and reaffirmed again in 2019, nations that signed onto the United Nation’s Sustainable Development Goals made the achievement of universal health coverage, including “access to safe, effective, quality and affordable essential medicines and vaccines for all,” a target of those SDG goals.^1^ But as countries work toward achieving universal health coverage, there is no consensus on how best to organize, fund, and manage health systems to meet that goal. The next set of authors interrogate the complex relationship between health systems and pharmaceutical access, considering different approaches for achieving universal health coverage for medicines, as well as different systems of pharmaceutical research and development, and the complexities and access barriers that emerge from those systems.

As Garcia et al. discuss in their essay, although Brazil’s public health system is premised on achieving universal health coverage for medicines, access to medicines remains a significant problem. For example, despite increased public funding for the provision of medicines, in 2014, there was no more than 62% availability of medicines in Brazil’s public healthcare system. As the authors note, the lack of access to medicine can induce increased costs elsewhere in the healthcare system, as untreated diseases lead to increased ambulatory care and hospitalization expenses. Governments, of course, have available to them different options for implementing the public provision of medicines. For example, they can pursue a strictly state-operated approach in which the public infrastructure of health care is used to finance and promote access to medicines. Or they can utilize public sector-private sector collaborative approach. In their article, Garcia et al. share the result of their economic analysis in which they compare the costs and access implications of two different approaches to funding and providing universal health coverage of medicines. The first model is representative of the current system in Brazil whereby the payment and logistic provision of medicines occurs entirely within the public healthcare system. The second model is that of a public-private sector collaboration in which private community pharmacies are accredited to dispense publicly-funded medicines. Both models retained the concept currently operational within Brazil’s NHS whereby there are no limits on medicines expenditures among families. Based on the results of their economic analysis, Garcia et al. propose an optimal private sector-public sector collaboration in which Brazilian citizens are referred to public health service centers in their neighborhood, where they receive medical care, prescriptions, and the authorization to obtain medicines in a private pharmacy of the citizen’s choice. Garcia et al. argue that this public-private collaboration “looks to be the key to achieve universal health coverage for medicines reducing avoidable hospitalization and mortality as well as inequalities among families concerning household expenses.” (p. 16).

In their article, Al-Hanawi et al. also explore the relationship between the public sector and private sector in government efforts to achieve universal health coverage. Focusing on the Kingdom of Saudi Arabia, Al-Hanawi et al. examine whether health insurance is an effective option for governments with public healthcare systems seeking to reduce out-of-pocket expenditures for health care as a potential way to increase the health and wellbeing of the country’s citizens through better access to medical care. As a high-income country with a mixed political economy of healthcare provision, the Kingdom of Saudi Arabia provides a compelling case study for addressing this question. Specifically, the Kingdom of Saudi Arabia provides free public healthcare to all public employees and to Saudi citizens. But Saudis who work in the private sector are required by law to receive employer-financed private health insurance. Using data from the 2018 Saudi Family Health Survey, the authors perform an econometric analysis to examine how health insurance relates to out-of-pocket expenditures on health and medicines along different levels of income. Their analysis shows that at low levels of income health insurance reduces out-of-pocket expenditures. They thus argue that “if the objective of policy is to cushion the relatively poor, then health insurance is a key.” (p. 8). However, as income levels rise, so too does the impact on out-of-pocket expenditures. Their findings also suggest that health insurance contributes to inequalities in both the quality of and access to healthcare. As such, Al-Hanawi et al. argue, policymakers in countries with public healthcare systems who are considering using health insurance as a means of insuring sustainability of healthcare financing while pursuing universal health coverage, need to consider “the possible welfare distribution impacts of upscaling or downscaling the coverage of insurance amongst the populations,” (pp. 1–2) as they pursue the goal of universal health coverage.

In his essay, Light calls for us to rethink the entire system of healthcare and pharmaceutical development. In the current market-driven system, “controlling disease through costly interventions creates or increases health disparities, as people with more knowledge, money, and beneficial social connections have greater access and ability to harness medical advances and treatment than those with less.” (p. 1). But as Light argues, the paradox of creating or increasing health disparities is not an inevitable outcome of controlling disease but rather the product of a neoliberal, market-based, inegalitarian society. Instead of leaving the research, development, and marketing of new pharmaceuticals and vaccines in the hands of private pharmaceutical companies, Light makes the case for non-profit health care and pharmaceutical development as a means of ameliorating health disparities. Light puts forth an alternative model, a “radical proposal” as he calls it, in which a virtual, non-profit, multi-stakeholder collaborative is responsible for researching, developing, testing, and manufacturing new, innovative, and affordable drugs that prioritizes treating those who are most disadvantaged and most ill. Light uses the Drugs for Neglected Diseases initiative (DNDi), particularly the DNDi’s success developing highly effective, low-cost drugs to eradicate Hepatitis C and creating markets to maximize public health rather than profits, as an example of his proposal in action. At the center of Light’s proposal (and the DNDi model) is the development of patent rights for public health, whereby patent and licensing powers are used to guarantee low prices and broad access at low profits.

As with any Research Topic covering almost limitless terrain, there are broad areas we were not able to cover. Among those specifically mentioned in our call for papers, we did not get articles covering the spectrum of pharmacology and indigenous systems of therapeutic knowledge; political economies and ecologies of sourcing and supplying pharmaceutical ingredients; the widespread problem of counterfeit and substandard drug trades; use of and impacts upon lab animals in the early testing of new drugs; the various means through which therapeutics are produced, paid for, sourced, and distributed; the growing field of alternative therapies and the politics of their regulation; or extended analyses of what Susan Reynolds-Whyte and others called ‘the social life of drugs’ (Whyte et al., 2006) - that is, the shift in relations among family members and communities resulting from having or lacking access to drugs.

The list could go on. And in part, this itself is evidence of the preeminent role pharmaceuticals play in our social, medical, political, financial, and quotidian lives. As such, this collection has from the beginning consciously limited our scrutiny to the importance of pharmaceuticals and the entangled terrains they create and occupy. It is worth noting, however, that equal attention needs to remain on what João Biehl has called ‘the pharmaceuticalization of health,’ that is, the over-reliance on pharmaceuticals in national healthcare systems, a practice that rests upon a dominant narrative of biotechnological capabilities and efficiencies in solving most health problems. We hope that in addition to this issue on pharmacology and global health, there will be future issues that interrogate what privileging pharmaceutical production and distribution does: what alternatives it might displace, or more salubrious futures might be possible with equal emphasis on other interventions. As COVID-19 has made so painfully clear, it is longstanding national or international inequities in wages, education, housing, nutrition, clean air and water, and other structural issues that determine health or its absence. The changes in government policies and global economic systems thus necessary to make more meaningful and lasting improvements in health have, so far, gained little traction. Rolling out COVID vaccines, though certainly life-saving and cause for celebration, will be equally effective in redirecting our collective attention away from the even more challenging social, systemic, economic, and political reconfiguring that is too long overdue. It will have to suffice at this point to acknowledge that we believe in the necessity of these broader changes and the ameliorative effects they could have on millions of lives, and to argue for further examination of those changes that are happening on smaller scales all over the world.

In the meantime, what our contributors have provided are incisive analyses of trenchant issues in the capacious arena of pharmaceutical access. Their optics have spanned theoretical frameworks, time periods, and geographies; and perhaps best of all, they have all raised at least as many questions as they have addressed, giving all of us more to think about and, hopefully, to share more insights in the future.

## Footnotes

1
https://www.un.org/sustainabledevelopment/health/
